# Factors associated with prostate specific antigen testing in Australians: Analysis of the New South Wales 45 and Up Study

**DOI:** 10.1038/s41598-018-22589-y

**Published:** 2018-03-09

**Authors:** Visalini Nair-Shalliker, Albert Bang, Marianne Weber, David E. Goldsbury, Michael Caruana, Jon Emery, Emily Banks, Karen Canfell, Dianne L. O’Connell, David P. Smith

**Affiliations:** 1 Cancer Research Division, Cancer Council NSW, Sydney, NSW Australia; 2 Sydney School of Public Health, Sydney Medical School, The University of Sydney, Sydney, NSW Australia; 3 Department of Clinical Medicine, Macquarie University, Sydney, Australia; 4 Department of General Practice, Centre for Cancer Research, University of Melbourne. Victorian Comprehensive Cancer Centre. Melbourne, Victoria, Australia; 5 National Centre for Epidemiology and Population Health, Research School of Population Health, Australian National University, Canberra, Australia; 6 School of Public Health and Community Medicine, University of New South Wales, Sydney, NSW Australia; 7 School of Medicine and Public Health, University of Newcastle, Newcastle, NSW Australia; 8 Menzies Health Institute, Queensland, Griffith University, Gold Coast, Queensland Australia

## Abstract

Australia has one of the highest incidence rates of prostate cancer (PC) worldwide, due in part to widespread prostate specific antigen (PSA) testing. We aimed to identify factors associated with PSA testing in Australian men without a diagnosis of prostate cancer or prior prostate disease. Participants were men joining the 45 and Up Study in 2006–2009, aged ≥45 years at recruitment. Self-completed questionnaires were linked to cancer registrations, hospitalisations, health services data and deaths. Men with a history of PC, radical prostatectomy or a “monitoring” PSA test for prostate disease were excluded. We identified Medicare reimbursed PSA tests during 2012–2014. Multivariable logistic regression was used to estimate adjusted odds ratios (OR) for the association between having PSA tests and factors of interest. Of the 62,765 eligible men, 51.8% had at least one screening PSA test during 2012–2014. Factors strongly associated with having a PSA test included having 27+ general practitioner consultations (versus 3–9 consultations; OR = 2.00; 95%CI = 1.90–2.11), benign prostatic hyperplasia treatment (versus none; OR = 1.59(95%CI = 1.49–1.70), aged 60–69 years (versus 50–59 years; OR = 1.54; 95%CI = 1.48–1.60). These results emphasise the important role of primary care in decision making about PSA testing.

## Introduction

Prostate cancer (PC) is the most common cancer diagnosed in men living in developed countries, after non-melanoma skin cancer^1^. Australia and New Zealand have some of the highest PC incidence rates in the world (111.6 new cases per 100,000), with an estimated 16,665 new cases predicted in Australian men in 2017^2,3^. Serum prostate specific antigen (PSA) is a test, used routinely as a *de facto* screening test for PC in asymptomatic males in many countries. Current evidence indicates that the benefit of population-wide PSA testing in averting death from PC is modest and the test itself is unable to distinguish indolent from aggressive cancers^4^. The subsequent harms associated with diagnosis and treatment, including risk of infection, bleeding and pain with biopsy, and urinary incontinence, erectile dysfunction and bowel problems associated with treatment mean that no jurisdiction internationally currently recommends population-wide programs for PC screening^5^.

In Australia, national clinical practice guidelines for PSA testing and early management of test-detected PC, released in 2016, recommend that for men at average risk of PC who have been informed of the benefits and harms of testing and who decide to undergo regular testing for PC, biennial PSA testing is conducted from age 50 to age 69 years^6^. Previous studies using data on self-reported PSA testing, age, family history of PC, health insurance status, visits to a doctor, and socioeconomic factors have been identified as potential predictors of PSA testing^7–10^, in Australia and other countries. However, the use of self-report of PSA testing has limitations as it may under-report its actual use.

Given the prevalence of PSA testing in Australia despite no systematic screening program, and the clinical and economic repercussions, it is important to understand what drives such testing and to understand how different testing behaviours in the population may relate to PC incidence. Our aim was to understand which factors are associated with having a PSA test for men without a PC diagnosis and with no prior monitoring of previous prostate disease. Using data from Medicare Australia reimbursements, we describe the proportion of men in a large population-based prospective study (the New South Wales (NSW) 45 and Up Study) who had one or more screening related PSA tests during 2012–2014, as this was the period with the most contemporaneous linked data, recognising that this may not be representative of PSA screening rates in the general population. We selected three years of testing data to enable comparisons to be drawn with the recent recommendations regarding biennial testing of informed men, and allowing for up to 12 months lag in testing compliance. We also examined the association of sociodemographic, behavioural, and health related factors with having a PSA test in this cohort.

## Results

Of the male participants without a history of PC and meeting other eligibility criteria, 51.8% had one or more Medicare reimbursed PSA tests, between 1^st^ January 2012 and 31^st^ December 2014. Crude proportions tested ranged from 41% of males aged 45–49 years through to 60% of those aged 60–69 and 30% of those aged 80 years and over. Compared to men aged 50–59 years the odds of having a PSA test was highest for men aged 60–69 years (OR = 1.54; 95%CI = 1.48–1.60) and lowest for those aged 80 years or greater (OR = 0.49; 95%CI = 0.46–0.52; Table 1).

**Table 1:** Sociodemographic characteristics associated with PSA testing in 2012–2014 for men in the NSW 45 and Up Study (n = 62,765).

Exposure	PSA^3^ testing			Minimal adjustment^1^				Full adjustment^2^			
	n (col %)	Yes (row %)	No (row %)	OR^3^	95% CI^3^		p-value	OR^3^	95% CI^3^		p-value
**Age at 2012**											
45–49	1157 (1.8)	474 (41.0)	683 (59.0)	—				0.66	0.58	0.74	<0.0001
50–59	22320 (35.6)	11373 (51.0)	10947 (49.0)	—				1.00			
60–69	19764 (31.5)	11910 (60.3)	7854 (39.7)	—				1.54	1.48	1.60	
70–79	11623 (18.5)	6344 (54.6)	5279 (45.4)	—				1.33	1.26	1.39	
80+	7901 (12.6)	2395 (30.3)	5506 (69.7)	—				0.49	0.46	0.52	
**Income** (**$AUD**)^3^											
Less than 20,000	10282 (16.4)	4522 (44.0)	5760 (56.0)	1.00			<0.0001	1.00			<0.0001
20,000–29,999	5780 (9.2)	2725 (47.1)	3055 (52.9)	1.09	1.02	1.16		1.09	1.02	1.16	
30,000–39,999	5148 (8.2)	2685 (52.2)	2463 (47.8)	1.25	1.16	1.34		1.25	1.16	1.34	
40,000–49,999	5133 (8.2)	2750 (53.6)	2383 (46.4)	1.30	1.21	1.39		1.31	1.22	1.40	
50,000–69,999	7795 (12.4)	4333 (55.6)	3462 (44.4)	1.40	1.32	1.49		1.41	1.33	1.51	
70,000 or more	20550 (32.7)	11412 (55.5)	9138 (44.5)	1.44	1.37	1.52		1.49	1.41	1.58	
Non respondents	8077 (12.9)	4069 (50.4)	4008 (49.6)	1.24	1.17	1.31		1.24	1.16	1.31	
**Qualification**											
No school certificate,any qualification	5877 (9.4)	2776 (47.2)	3101 (52.8)	1.00			<0.0001	1.00			<0.0001
School or intermediate certificate	9192 (14.6)	4741 (51.6)	4451 (48.4)	1.16	1.08	1.24		1.11	1.04	1.19	
Higher school or leaving certificate	6309 (10.1)	3254 (51.6)	3055 (48.4)	1.15	1.07	1.23		1.06	0.98	1.14	
Trade or apprenticeship	11791 (18.8)	6241 (52.9)	5550 (47.1)	1.23	1.15	1.31		1.17	1.09	1.24	
Certificate or diploma	12448 (19.8)	6554 (52.7)	5894 (47.3)	1.19	1.11	1.27		1.07	1.00	1.15	
University degree or higher	17148 (27.3)	8930 (52.1)	8218 (47.9)	1.15	1.08	1.22		0.98	0.92	1.05	
**Place of residence**											
Major Cities	33606 (53.5)	17449 (51.9)	16157 (48.1)	1.00			<0.0001	1.00			0.0153
Inner Regional	21867 (34.8)	11323 (51.8)	10544 (48.2)	0.94	0.91	0.98		0.96	0.93	0.99	
Outer Regional, Remote,Very Remote	7292 (11.6)	3724 (51.1)	3568 (48.9)	0.90	0.86	0.95		0.94	0.89	0.99	
**Married or living with a partner**											
No	11512 (18.3)	5067 (44.0)	6445 (56.0)	1.00			<0.0001	1.00			<0.0001
Yes	51253 (81.7)	27429 (53.5)	23824 (46.5)	1.39	1.34	1.45		1.31	1.26	1.37	
**Health cover**											
None	10808 (17)	5166 (47.8)	5646 (52.2)	1.00			<0.0001	1.00			<0.0001
DVA^3^/Health care concession card	11272 (17.8)	4469 (39.6)	6828 (60.6)	0.79	0.75	0.83		0.81	0.76	0.86	
Private health insurance	41380 (65.2)	23176 (56)	18226 (44)	1.40	1.34	1.46		1.41	1.35	1.48	
**Region of Birth**											
Australia	46280 (73.7)	24233 (52.4)	22047 (47.6)	1.00			<0.0001	1.00			<0.0001
North America	464 (0.7)	214 (46.1)	250 (53.9)	0.73	0.61	0.88		0.73	0.61	0.89	
United Kingdom or Ireland	6658 (10.6)	3216 (48.3)	3442 (51.7)	0.86	0.81	0.90		0.85	0.80	0.89	
New Zealand	1291 (2.1)	648 (50.2)	643 (49.8)	0.90	0.80	1.00		0.88	0.79	0.99	
Oceania	212 (0.3)	99 (46.7)	113 (53.3)	0.80	0.61	1.05		0.80	0.61	1.06	
South Asia	448 (0.7)	213 (47.5)	235 (52.5)	0.84	0.70	1.02		0.85	0.70	1.03	
Far East Asia	793 (1.3)	397 (50.1)	396 (49.9)	0.94	0.82	1.08		0.99	0.85	1.14	
Western Europe	2985 (4.8)	1557 (52.2)	1428 (47.8)	1.02	0.94	1.10		1.03	0.95	1.11	
Africa	531 (0.8)	286 (53.9)	245 (46.1)	1.05	0.89	1.25		1.03	0.87	1.23	
South East Asia	1046 (1.7)	552 (52.8)	494 (47.2)	1.02	0.90	1.16		1.07	0.94	1.21	
Central Europe	835 (1.3)	421 (50.4)	414 (49.6)	1.06	0.92	1.22		1.09	0.94	1.25	
North Africa and Middle East	588 (0.9)	313 (53.2)	275 (46.8)	1.02	0.86	1.20		1.09	0.92	1.29	
Central America	267 (0.4)	153 (57.3)	114 (42.7)	1.14	0.89	1.46		1.16	0.91	1.49	
Unspecified countries	367 (0.6)	194 (52.9)	173 (47.1)	1.10	0.89	1.35		1.12	0.91	1.38	

^1^Model includes age and each factor individually so ORs are adjusted for age only.

^2^Model includes age, place of residence, qualification, income and each additional factor individually.

^3^Abbreviations: AUD, Australian dollar; CI, confidence interval; DVA, Department of Veteran Affairs; NSW, New South Wales; OR, odds ratio; PSA, prostate specific antigen.

### Demographic factors

The proportions who had a PSA test increased with increasing household income: OR for highest compared with lowest group (OR = 1.49; 95%CI: 1.41–1.58), while men living in inner regional areas (OR = 0.96; 95%CI: 0.93–0.99) or in outer regional or remote areas (OR = 0.94; 95%CI: 0.89–0.99), had lower odds of being tested compared to those living in major cities. The odds of having a PSA test were higher for men with higher levels of education compared to those with no school certificate or any qualification, men who were married or living with a partner (versus not married or single), or for men who had private health insurance or concession card holders (versus no private health insurance). The region in which men were born was associated with testing (P < 0.0001), with men born in North America (OR = 0.73; 95%CI = 0.61–0.89), United Kingdom (OR = 0.85; 95%CI = 0.80–0.89) or New Zealand (OR = 0.88; 95%CI: 0.79–0.99) having lower odds of having a screening PSA test, compared to Australian-born men.

### Health service factors

Each of the four health service use and health related factors examined in Table 2 were significantly associated with having a PSA test. Only 14% of men who never or rarely visited a GP in the study period had a test in the study period. The odds of testing increased with increasing GP visits, (OR = 2.00; 95%CI: 1.90–2.11) in men who visited a GP at least 27 times in the 3 years compared to men who visited a GP 3–9 times. Men who took more supplements or medications had higher odds of testing. Men who reported having a faecal occult blood test (vs. no test; OR = 1.32; 95%CI: 1.27–1.37) or who reported having at least one non-prostate related medical condition had higher odds of having a PSA test.

**Table 2:** Association between health services use, health related factors and PSA testing in 2012–2014 for men in the NSW 45 and Up Study (n = 62,765).

Exposure	PSA testing PSA Screening test			Minimal adjustment^1^				Full adjustment^2^			
	n (col %)	Yes (row %)	No (row %)	OR^3^	95% CI^3^		p-value	OR	95% CI^3^		p-value
**GP** ^3^ **visits frequency**											
0–2	5937 (9.5)	856 (14.4)	5081 (85.6)	0.21	0.20	0.23	<0.0001	0.22	0.20	0.23	<0.0001
3–9	14650 (23.3)	7022 (47.9)	7628 (52.1)	1.00				1.00			
10–16	14915 (23.8)	8610 (57.7)	6305 (42.3)	1.49	1.43	1.57		1.51	1.44	1.59	
17–26	13075 (20.8)	7852 (60.1)	5223 (39.9)	1.77	1.68	1.86		1.83	1.74	1.93	
27+	14188 (22.6)	8156 (57.5)	6032 (42.5)	1.84	1.75	1.94		2.00	1.90	2.11	
**Supplementation or medication use**											
0	12015 (19.1)	5816 (48.4)	6199 (51.6)	1.00			<0.0001	1.00			<0.0001
1	12191 (19.4)	6366 (52.2)	5825 (47.8)	1.21	1.15	1.27		1.22	1.16	1.28	
2	11637 (18.5)	6249 (53.7)	5388 (46.3)	1.31	1.24	1.38		1.32	1.25	1.39	
3 and over	22314 (35.6)	11744 (52.6)	10570 (47.4)	1.35	1.29	1.41		1.38	1.31	1.44	
Yes but unknown	4608 (7.3)	2321 (50.4)	2287 (49.6)	1.13	1.05	1.21		1.15	1.07	1.23	
**Bowel screening** (**FOBT**)^3^											
No	45213 (72.0)	22359 (49.5)	22854 (50.5)	1.00			<0.0001	1.00			<0.0001
Yes	17552 (28.0)	10137 (57.8)	7415 (42.2)	1.33	1.28	1.38		1.32	1.27	1.37	
**Non-prostate medical conditions**											
0 medical condition (None of these)	13689 (21.8)	6897 (50.4)	6792 (49.6)	1.00			<0.0001	1.00			<0.0001
1 medical condition	23067 (36.8)	12257 (53.1)	10810 (46.9)	1.15	1.10	1.20		1.16	1.11	1.21	
2 medical conditions	16895 (26.9)	8933 (52.9)	7962 (47.1)	1.21	1.16	1.27		1.23	1.18	1.29	
3 medical conditions	7422 (11.8)	3594 (48.4)	3828 (51.6)	1.08	1.01	1.14		1.12	1.05	1.19	
No medical condition selected	1692 (2.7)	815 (48.2)	877 (51.8)	0.94	0.85	1.04		0.95	0.86	1.06	

^1^OR adjusted for age. ^2^OR adjusted for age, place of residence, qualification, and income.

^3^Abbreviations: CI, confidence interval; OR, odds ratio; GP, general practitioner; FOBT, faecal occult bowel testing.

Reporting poorer overall health, lower quality of life, or higher psychosocial distress at baseline, were associated with a lower odds of PSA testing (Table 3). Compared to men who reported having excellent overall health or quality of life, those who reported poor status had lower odds of having had a PSA test (OR = 0.61; 95%CI: 0.54–0.70 and OR = 0.56; 95%CI: 0.49–0.64 respectively). Those with severe psychological distress had lower odds of having a PSA test (OR = 0.69; 95%CI: 0.61–0.78) compared to men with lower K10 psychological distress scores.

**Table 3:** Association between psychosocial status and PSA testing in 2012–14 for men in the NSW 45 and Up Study (n = 63,616).

Exposure	PSA testing			Minimal adjustment^1^				Full adjustment^2^			
	n (col %) 63616	Yes (row %)	No (row %)	OR	95% CI		p-value	OR	95% CI		p-value
**Overall health**											
Excellent	9047 (14.4)	4794 (53.0)	4253 (47.0)	1.00			<0.0001	1.00			<0.0001
Very good	23371 (37.2)	12834 (54.9)	10537 (45.1)	1.11	1.06	1.17		1.12	1.07	1.18	
Good	21861 (34.8)	11146 (51.0)	10715 (49.0)	0.99	0.95	1.04		1.02	0.97	1.07	
Fair	7300 (11.6)	3280 (44.9)	4020 (55.1)	0.80	0.75	0.85		0.85	0.80	0.91	
Poor	1186 (1.9)	442 (37.3)	744 (62.7)	0.55	0.48	0.62		0.61	0.54	0.70	
**Quality of life**											
Excellent	14265 (22.7)	7819 (54.8)	6446 (45.2)	1.00			<0.0001	1.00			<0.0001
Very good	24211 (38.6)	13199 (54.5)	11012 (45.5)	1.03	0.99	1.07		1.03	0.99	1.08	
Good	17950 (28.6)	8858 (49.3)	9092 (50.7)	0.89	0.85	0.93		0.91	0.87	0.95	
Fair	5299 (8.4)	2236 (42.2)	3063 (57.8)	0.67	0.63	0.72		0.72	0.67	0.77	
Poor	1040 (1.7)	384 (36.9)	656 (63.1)	0.51	0.44	0.58		0.56	0.49	0.64	
**Psychological distress** (**K10**)											
Well (10 - lt 20)	52100 (83.0)	27590 (53.0)	24510 (47.0)	1.00			<0.0001	1.00			<0.0001
Mild (20 - lt 25)	3435 (5.5)	1668 (48.6)	1767 (51.4)	0.82	0.76	0.88		0.86	0.80	0.92	
Moderate (25 - lt 30)	1122 (1.8)	493 (43.9)	629 (56.1)	0.67	0.60	0.76		0.72	0.64	0.82	
Severe (30–50)	1054 (1.7)	444 (42.1)	610 (57.9)	0.62	0.54	0.70		0.69	0.61	0.78	
UNSP	5054 (8.1)	2301 (45.5)	2753 (54.5)	0.89	0.84	0.94		0.92	0.87	0.98	

^1^OR adjusted for age. ^2^OR adjusted for age, place of residence, ^1^OR adjusted for age. ^2^OR adjusted for age, place of residence, income.

### Prostate cancer related factors

We examined a selection of established or potential risk factors of PC in our analysis (Table 4). Compared to those with no family history of cancer, the odds of having a PSA test increased for those having a family history of only PC (OR 1.22; 95%CI% = 1.14–1.31), PC and breast cancer (OR 1.33; 95%CI = 1.16–1.52), or PC and other cancers (OR 1.21; 95%CI = 1.09–1.34), while there was no difference between those with a family history of cancer other than PC. Men had higher odds of having a PSA test if they had been prescribed BPH medication (vs. non-users; OR 1.59; 95%CI = 1.49–1.70), were diabetic (vs. non-diabetics; OR = 1.11; 95%CI = 1.06–1.15), had a vasectomy (vs. none; OR 1.18; 95%CI = 1.14–1.23), or were overweight (vs. normal weight; OR 1.18 95%CI: 1.13–1.22) or obese (vs. normal; OR 1.15; 95%CI = 1.10–1.20). Of men with a family history of prostate cancer, aged between 45 and 49 years, 45.5% had a PSA test record (data not shown). In terms of health behaviours, men who consumed any amounts of alcohol (vs. none), or who participated in more than 3 physical activity sessions per week (vs. <3 sessions per week), or reported urinary bother, all had higher odds of having had a PSA test. The proportion who had a PSA test was lower for men who were ever smokers (vs. never smokers; OR = 0.89; 95%CI: 0.86–0.92), and men who reported erectile dysfunction (vs. none; OR 0.94 95%CI: 0.90–0.98).

**Table 4:** Association between established and potential prostate cancer risk factors and PSA testing in 2012–2014 for men in the NSW 45 and Up Study (n = 63,616).

Exposure	PSA testing			Minimal adjustment^1^				Full adjustment^2^			
	n (col %)	Yes (row %)	No (row %)	OR	95% CI		p-value	OR	95% CI		p-value
**Parental history of cancer**											
No cancer	36566 (58.3)	18605 (50.9)	17961 (49.1)	1.00			<0.0001	1.00			<0.0001
PC only (No other Cn)	3674 (5.9)	2078 (56.6)	1596 (43.4)	1.23	1.14	1.31		1.22	1.14	1.31	
PC and BC/ovarian only and/or other cancer	896 (1.4)	516 (57.6)	380 (42.4)	1.32	1.15	1.51		1.33	1.16	1.52	
PC and other cancer (Non- BC/ovarian) breast/ovarian)	1640 (2.6)	921 (56.2)	719 (43.8)	1.21	1.10	1.34		1.21	1.09	1.34	
BC/OvC (No PC)	6782 (10.8)	3528 (52.0)	3254 (48.0)	1.03	0.97	1.08		1.02	0.97	1.08	
Other cancer	13207 (21.0)	6848 (51.9)	6359 (48.1)	1.02	0.98	1.06		1.01	0.97	1.06	
**BPH medication**											
No	58332 (92.9)	29966 (51.4)	28366 (48.6)	1.00			<0.0001	1.00			<0.0001
Yes	4433 (7.1)	2530 (57.1)	1903 (42.9)	1.51	1.41	1.61		1.59	1.49	1.70	
**Diabetes**											
No	48570 (77.4)	24986 (51.4)	23584 (48.6)	1.00			0.0004	1.00			<0.0001
Yes	14195 (22.6)	7510 (52.9)	6685 (47.1)	1.07	1.03	1.12		1.11	1.06	1.15	
**Vasectomy**											
No	46193 (73.6)	23067 (49.9)	23126 (50.1)	1.00			<0.0001	1.00			<0.0001
Yes	16572 (26.4)	9429 (56.9)	7143 (43.1)	1.21	1.17	1.26		1.18	1.14	1.23	
**Erectile dysfunction**											
No	38903 (62.0)	21287 (54.7)	17616 (45.3)	1.00			<0.0001	1.00			<0.0001
Yes	17859 (28.5)	8559 (47.9)	9300 (52.1)	0.90	0.87	0.94		0.94	0.90	0.98	
Missing/Not answered	6003 (9.6)	2650 (44.1)	3353 (55.9)	0.78	0.73	0.82		0.81	0.76	0.86	
**Body mass index** (**BMI**)											
Under weight	421 (0.7)	138 (32.8)	283 (67.2)	0.57	0.46	0.70	<0.0001	0.60	0.48	0.74	<0.0001
Normal weight	18573 (29.6)	8849 (47.6)	9724 (52.4)	1.00				1.00			
Overweight	29418 (46.9)	15792 (53.7)	13626 (46.3)	1.19	1.15	1.24		1.18	1.13	1.22	
Obese	14353 (22.9)	7717 (53.8)	6636 (46.2)	1.14	1.09	1.20		1.15	1.10	1.20	
**Weekly alcohol consumption**											
0	14045 (22.4)	6706 (47.7)	7339 (52.3)	1.00			<0.0001	1.00			<0.0001
1–6 (inclusive)	17098 (27.2)	8906 (52.1)	8192 (47.9)	1.15	1.10	1.20		1.11	1.06	1.16	
7–20 (inclusive)	22721 (36.2)	12216 (53.8)	10505 (46.2)	1.23	1.17	1.28		1.17	1.12	1.22	
21+	8901 (14.2)	4668 (52.4)	4233 (47.6)	1.10	1.04	1.16		1.06	1.00	1.12	
**Physical activity** (**Sessions per week**)											
0–3	9209 (14.7)	4424 (48.0)	4785 (52.0)	1.00			<0.0001	1.00			<0.0001
4–6	9725 (15.5)	5169 (53.2)	4556 (46.8)	1.20	1.13	1.27		1.18	1.12	1.25	
7–10	14767 (23.5)	7823 (53.0)	6944 (47.0)	1.20	1.14	1.26		1.18	1.12	1.25	
11–17	15259 (24.3)	7965 (52.2)	7294 (47.8)	1.14	1.09	1.21		1.13	1.07	1.19	
18+	13805 (22.0)	7115 (51.5)	6690 (48.5)	1.08	1.02	1.14		1.08	1.02	1.14	
**Urinary bother**											
Mild (0–5)	46303 (73.8)	24208 (52.3)	22095 (47.7)	1.00			0.0298	1.00			0.002
Moderate (6–11)	8771 (14.0)	4561 (52.0)	4210 (48.0)	1.07	1.02	1.13		1.09	1.04	1.14	
Severe (12–21)	1506 (2.4)	777 (51.6)	729 (48.4)	1.04	0.94	1.16		1.10	0.99	1.23	
Missing	6185 (9.9)	2950 (47.7)	3235 (52.3)	1.00	0.95	1.06		1.03	0.98	1.09	
**Smoking status**											
Never	30521 (48.6)	16273 (53.3)	14248 (46.7)	1.00			<0.0001	1.00			<0.0001
Ever	32244 (51.4)	16223 (50.3)	16021 (49.7)	0.88	0.85	0.91		0.89	0.86	0.92	

^1^OR is adjusted for age.

^2^OR adjusted for age, place of residence, qualification, and income.

## Discussion

This large study showed that a high proportion of middle aged and older PC free Australian men underwent PSA testing during a three year period. Our results identified socio-economic and health system factors associated with having a PC test. Increasing frequency of GP visits, treatment for BPH or being aged 60–69 years were associated with at least 50% higher odds of PSA testing during the study period. Private health insurance, high household income, lower levels of psychological distress, better overall quality of life, higher supplement use, being married, a history of bowel screening and having a family history of prostate and breast or ovarian cancers were associated with increased odds of having a PSA test of between 30% and 49%. The odds of having at least one PSA test, in this study period, were up to 20% higher or lower than referent groups for factors such as region of birth, vasectomy, physical activity, obesity, smoking, diabetes, and erectile dysfunction.

Our finding that the frequency of GP visits was associated with higher PSA testing is consistent with other studies showing men who did not have a routine health check-up or lacked access to health care were less likely to have a PSA test^7,11^. PSA testing occurs principally in the primary care setting and it is therefore reasonable to predict that the more interaction men have with a GP the more opportunity there is to be tested. We found that a number of health related factors were associated with higher testing, including previous bowel cancer screening, supplement and medication use, a diagnosis of BPH, diabetes, history of vasectomy, urinary bother and reporting a higher number of other medical conditions. In the context of men with a diagnosis of BPH or reporting urinary bother, it is possible that the PSA test was conducted as part of their clinical assessment and therefore not strictly speaking as a screening test. However, not all health related factors that may lead to higher patient interaction with a GP were positively associated with higher testing rates. Notable exceptions identified in this study were for men reporting worse overall health, lower quality of life or more severe psychological distress. Ever smokers or men with self-reported erectile dysfunction, had lower odds of having had a PSA test than other men. Few studies have assessed the relationship between psychological well-being and PC testing. A recent US study showed men with higher perceived stress were less likely to report a PSA test but that anxiety appeared to influence testing through the interaction with the number of GP visits men had^12^. However there was a time gap between completing the self-report measures for psychological distress and the event of PSA testing in our study. It is possible that GPs are more likely to forego tests on these men who may have remained distressed all throughout the follow up period, or have previously experienced distress, as the instigation of testing and the series of events that may follow may increase psychological burden for men with higher levels of stress. It may also reflect the limited time in a primary care consultation and deliberate focus of attention on mental health issues instead of PSA testing.

Married or partnered men had higher odds of being tested than single or unmarried men reflecting similar findings from other studies^12–14^. Spousal encouragement to attend PSA testing has previously been documented in a high-risk Australian cohort^15^, and is also likely to play a role particularly when partners are engaged in breast and cervical screening programs. Previous studies have reported that men with no health insurance were less likely to have a PSA test and this is consistent with our findings^7,11^. However, unlike the USA, in Australia an annual PSA test in men with no prior prostate disease is funded by Medicare, the universal healthcare system, and therefore the association with health insurance status in our study is less likely related to access to the test and more likely a proxy for engagement with health care services.

We found that uptake of PSA testing differed by age. Compared to men in their fifties, the odds of having a PSA test was highest for men aged between 60 and 69 years followed by those aged between 70 and 79 years. It is worth noting that 41% of men in their 40 s, 55% of men in their 70 s and 30% of those aged 80+ years had a PSA test. This compares with estimates reported in the USA where approximately one third of men aged over 75 were screened in 2015^16^. This high rate of testing for men aged below 50 and above 70 years in the absence of any evidence for a benefit in this population is concerning. Although our analysis period pre-dates the introduction of the 2016 Australian guidelines for PSA testing, the finding of the peak in testing rates for men aged 50–69 is partly consistent with the recommendations which state that for men who are fully informed of the benefits and harms and decide to test, that biennial testing from 50 years up to 69 years is suggested^6^.

The Australian PSA testing guidelines also recommend that fully informed testing should commence at 45 years of age only for men with a family history of PC. While forty one per cent of men reporting a family history in this age group had a PSA test only 10% of the whole cohort reported a family history of PC. We observed that men with a self-reported family history of PC had a higher odds of having a PSA test, compared to those with no family history of any cancer, and that the highest odds were in men with a family history of prostate and breast or ovarian cancer. This relationship is consistent with a case control study of ~600 men from Queensland, Australia^8^.

Men living in regional and remote areas had lower odds of having a PSA test than residents of major cities. A recent analysis of Australian Medicare data showed that men resident in very remote Australia were 43% less likely to have had a PSA test than their urban counterparts (*Calopedos under review)*. We found a much smaller difference, perhaps as a consequence of the selection of the participants in the 45 and Up Study, who appear to be more engaged with the health system (healthy screeners) than the general population and have higher odds of being tested. However, this may not simply be a patient driven phenomenon. A survey of over 400 GPs across Australia, reported that more doctors from metropolitan areas requested PSA tests compared to doctors from rural areas^17^.

The strong association between prescriptions for BPH treatment and PSA testing is consistent with men who are prescribed these medications having a PSA test as part of a clinical assessment of lower urinary tract symptoms. Our exclusion of men with a Medicare item number 66656 used to categorise claims for men who are ‘monitoring’ their prostate health, was designed to exclude most men with symptoms for BPH, or any other prostate problems. A cross tabulation between claims for BPH prescription medicines and self-reported enlarged prostate (data not shown) showed that 66% of the men with BPH prescription medicines (n = 4505) did not report BPH at baseline suggesting that these men may not have had BPH at the time of recruitment but may have since developed it. A cross tabulation between claims for BPH prescription medicines and self-reported urinary bother showed that only 4%, 15% and 26% of men reported having mild, moderate or severe lower urinary tract symptoms respectively at baseline were prescribed BPH prescription medicines in the study period.

A diagnosis of diabetes was associated with greater odds of having a PSA test. We used data on the prescription of diabetic medications to identify diabetics, a method shown to have a positive predictive value of 85% in predicting incidence of diabetes^18^. The associations between diabetes and PC appear complex. An analysis of over 9000 participants from 5 nations (including Australia), showed a reduced risk of developing PC for diabetic men (HR 0.56; 95%CI: 0.51, 0.61) and this risk decreased further with increased diabetes duration^19^. A 10 year follow-up study of over 1 million men with no previous history of cancer, reported that men with diabetes were more frequently tested than those without diabetes but the proportion of those with PSA levels above 4ng/mL was lower for men with diabetes than those without^20^. This may reflect the addition of a PSA test at a time when blood is drawn for monitoring other non-prostate related medical conditions.

In addition to its size and longitudinal design, the major strength of this study lies in the use of linked population-wide administrative data to prospectively identify PSA testing. Previous studies of PSA testing have predominantly used self-report as an outcome which has been shown to be subject to considerable measurement error and generally appears to under-enumerate true testing recall^21^. We excluded men based on prior reasons for PSA tests and if they had been diagnosed with PC or prostate diseases, therefore making the outcome for this analysis a more focussed investigation of screening asymptomatic men.

The study sample is reasonably representative of the NSW population, comprising approximately 10% of the total population aged 45 and over, however the response rate for the 45 and Up Study is relatively modest, at 18%^22^. Similar to other cohort studies, participants in the 45 and Up Study are a generally healthier and may be more engaged in testing and screening behaviours than the general population. However, the observed approximate 50% testing rate during the three years of the study is similar to self-reported testing rates from other Australian studies^23^. Importantly, the associations quantified here are based on internal comparisons and methodological work shows that such comparisons are valid even though the cohort is not fully representative of the target population^12^. A limitation of using Medicare data is that it was not possible to know the clinical indication for performing the test. Also not all PSA tests are recorded in the Medicare database as tests on public patients in public hospitals are not captured. In addition episode coning occurs when more than three items are requested in an episode by a GP for an out-of-hospital service and Medicare Australia only pays for, and therefore records the three most expensive items. It is estimated that approximately 7.5% of the Medicare claims for PSA tests may be subjected to episode coning^24^.

These results are important for two reasons. Firstly, in a time when peak bodies are evaluating and revising guidelines for the early detection and treatment of PC it is essential to know which sub-groups of the population are already engaged in population wide testing^6,16^. Australian guidelines recommended testing be ceased after age 70 and only offered to men below 50 if they are at increased risk of PC. We found that significant proportions of these age groups were being tested. Understanding the socio-demographic and health-related characteristics of this group of men, who are unlikely to benefit from testing, is a necessary next step. Secondly, it is important to understand relationships between testing behaviours and risk of PC. A number of the characteristics associated with higher odds of PSA testing (family history, weight, vasectomy) reported here have also been shown to be associated with the risk of PC. Australia has one of the highest incidence rates for PC internationally, due in part to widespread *ad hoc* PSA testing. It is likely that testing patterns in certain groups have driven higher incidence rates and conversely lower testing in groups who are unlikely to experience long term benefit may results in lower incidence rates and appear to convey artefactual protective effects. A better understanding of how this might affect epidemiological studies investigating associations between these risk factors and stage and grade of PC, is needed. Our data do not allow us to comment on how well men were informed about PSA testing. Given the high rates of testing and concerns about the balance between benefits and harms of the test, the issue remains about ensuring men are supported in making informed decisions regarding PC testing.

## Methods

### Participants

The Sax Institute’s 45 and Up Study is a large NSW population-based prospective study of male and female participants aged 45 years and above, designed to investigate healthy ageing^25^. Of the 267,019 participants recruited, 123,882 were males. All participants were enrolled between 2006 and 2009. Participants were randomly sampled from the Department of Human Services (formerly Medicare Australia) enrolment database, a national publically funded universal health care system which covers all citizens, permanent residents and some temporary residents and refugees. Those aged over 80 years and residents of regional areas were over-sampled by a factor of two. All participants completed a postal questionnaire at recruitment which included information on sociodemographic factors, health behaviours, and medical history, and provided consent for linkage of their data to population health databases. Study methods and a characterisation of the cohort, described in detail elsewhere, show that the exposure-outcome relationships for a broad range of factors were consistent with those observed in the NSW Public Health Survey^22^.

### Ethical approval

Ethics approval for the 45 and Up Study was provided by the University of NSW Human Research Ethics Committee and for this analysis of PSA testing behaviour by the NSW Population and Health Services Research Ethics Committee (HREC/14/CIPHS/54). The use of Medicare and Pharmaceutical Benefits Scheme (PBS) data for the purpose of this analysis was approved by the Department of Health Departmental Ethics Committee. All participants provided informed consent to the use and linkage of their data. All methods were performed in accordance with the relevant guidelines and regulations governing the use of these data.

### Data linkage

In 2015, records of all participants were linked with selected administrative health datasets, by the Centre for Health Record Linkage (CHeReL). These datasets were; NSW Cancer Registry (NSWCR: Jan 1994–Dec 2010), Cause of Death Unit Record File (CODURF: Feb 2006–Dec 2012), Registry of Births, Deaths and Marriages (RBDM: Feb 2006–Dec 2014), NSW Admitted Patient Data Collection (APDC: Jul 2001–Jun 2014). Probabilistic record linkage with these administrative health databases provided information on participants’ cancer diagnoses, death, and diagnoses and procedures during hospital admissions. In addition, the Sax Institute used a unique identifier to conduct deterministic linkage with claims made to the Medicare Benefits Scheme (MBS: Jun 2004–Dec 2014) and Pharmaceutical Benefits Schedule (PBS: Jun 2004–Dec 2014) data provided by the Department of Human Services. This provided information on PSA tests, General Practitioner (GP) visits and use of finasteride and prescribed medicines for diabetes.

For the purposes of this study we selected the most recent three year period (1/1/2012–31/12/2014) for which linked data were available. Men were classified as having had a PSA “screening” test if one or more claims were made for the related MBS Item (Item #66655) during 2012–2014.

### Exclusion criteria

**Figure 1:**
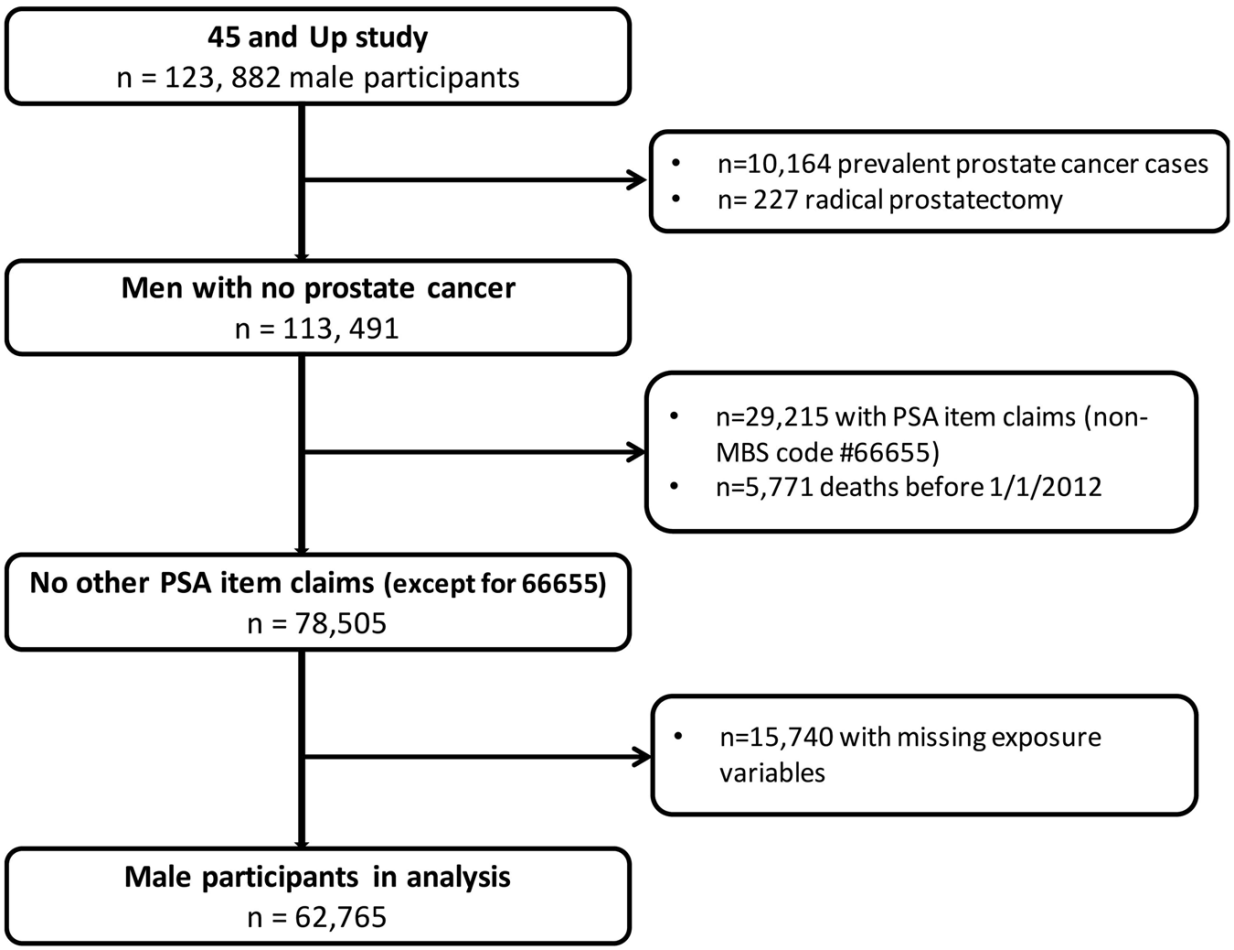
Flow diagram showing final derivation of participants from the original 45 and Up Study cohort.

Men meeting the following criteria were excluded to ensure only those without a history of PC was included in this analysis (illustrated in Fig. 1):

(i) Men with a PC diagnosis (ICD-10 C61) prior to 1/1/2012 registered by the NSWCR or with APDC records listing C61 diagnosis codes (n = 10,164) or a radical prostatectomy recorded in Medicare Benefits Schedule (MBS) claims prior to 1/1/2012 (n = 227).
(ii) Men with a Medicare record for any reimbursements for PSA test for monitoring “previous prostatic disease” or a follow up of a previously elevated test (MBS item numbers: 66656, 66659, 66660; n = 29,215), between 1/1/2012 and 31/12/2014.
(iii) Men who were registered as having died before 1/1/2012 as recorded in the NSWCR, CODURF or RBDM (n = 5,771).
(iv) Those with missing information for a single variable for which ≥10% of values were missing, were included in the analysis as “missing”.

### Coding and categorisation

Sociodemographic characteristics were grouped as follows and categories are listed in Tables 1 and 2: Age on 1/1/2012; Annual household income; highest level of education at study recruitment; Place of residence at recruitment using the Accessibility Remoteness Index of Australia (ARIA+) which was derived from the residential postcode; Marital status at recruitment; Health insurance cover; Region of birth was grouped according to the Global Burden of Disease Study.

Frequency of visits to a general practitioner (GP) between 1/1/2012 and 31/12/2014, was ascertained from MBS claims and categorized into 0–2 visits and the remaining numbers of visits were divided into quartiles; Bowel cancer screening was self-reported at recruitment as ever having a Faecal Occult Blood Test (FOBT); which was the inception of initial rollout of Australia’s National Bowel Cancer Screening Program and thus is likely to predominantly reflect elective testing, Medical conditions and use of supplementations or medications were self-reported at recruitment.

Participant’s psychosocial status was based on the level of psychological distress (based on Kessler Psychological Distress Scale), and overall self-rated health and quality of life, reported at recruitment.

PC risk factors were examined and grouped accordingly: family history of cancer in first degree relatives; treatment of benign prostatic hyperplasia was obtained from PBS records (Items for prazosin, terazosin, doxazosin, tamsulosin and finasteride) for prescriptions between 1/1/2004 to 31/12/2014; Incidence of diabetes was determined from PBS (ATC code – A10), MBS and APDC records for prescriptions between 1/1/2004 to 31/12/2014, previously described^18^.

The following variables were derived using self-reported information at recruitment: Body Mass Index (BMI) based on height (m) and weight (kg), was categorized using World Health Organisation classification; number of weekly alcoholic drinks where one drink was either “a glass of wine, middy of beer or nip of spirit”, was categorised as non-drinkers (0), and then divided into tertiles; Physical activity derived from frequency of vigorous and non-vigorous physical activity sessions undertaken in a week and categories were developed as previously described^26^. Total number of sessions was weighted against the intensity of the workout, where vigorous exercise was given twice the weight of less vigorous exercise, giving rise to the total number of exercise sessions the participant engaged in during a normal week; dichotomous variables were derived for smoking status, and vasectomy. Erectile dysfunction was categorized as ‘No’ for those who reported that they always or usually get and keep an erection firm enough for satisfactory sexual activity, ‘Yes’ for those who reported that this happened only sometimes, or never and ‘missing/not answered’. Urinary bother was derived from the modified IPSS (m-IPSS) and categorized as 0–5 (no/mild symptoms), 6–11 (moderate symptoms), 12–21 (severe symptoms) and missing.

Odds ratios (ORs) and 95% confidence intervals (CI) for having a PSA test and each of the factors of interest were estimated using multivariable logistic regression, first adjusting for age, and then with further adjustment for age, place of residence, education and household income. All analyses were carried out in SAS version 9 (SAS Institute Inc., Cary, NC, US).

### Data availability statement

The authors confirm that, for approved reasons, some access restrictions apply to the data underlying the findings. We obtained the data for the project from a third party, namely the Sax Institute, which is the data custodian for the 45 and Up Study. Data are available through application to the Sax Institute. Details are available at https://www.saxinstitute.org.au/our-work/45-up-study/ or through contacting 45andUp.research@saxinstitute.org.au.
